# Is Consumer Response to Plain/Standardised Tobacco Packaging Consistent with Framework Convention on Tobacco Control Guidelines? A Systematic Review of Quantitative Studies

**DOI:** 10.1371/journal.pone.0075919

**Published:** 2013-10-16

**Authors:** Martine Stead, Crawford Moodie, Kathryn Angus, Linda Bauld, Ann McNeill, James Thomas, Gerard Hastings, Kate Hinds, Alison O'Mara-Eves, Irene Kwan, Richard I. Purves, Stuart L. Bryce

**Affiliations:** 1 Institute for Social Marketing & Cancer Research United Kingdom Centre for Tobacco Control Research and United Kingdom Centre for Tobacco and Alcohol Studies, University of Stirling, Stirling, United Kingdom; 2 Addictions Department, Institute of Psychiatry, King's College London, United Kingdom Centre for Tobacco and Alcohol Studies, London, United Kingdom; 3 Evidence for Policy and Practice Information and Co-ordinating-Centre, Social Science Research Unit, Institute of Education, London, United Kingdom; The University of Adelaide, Australia

## Abstract

**Background and Objectives:**

Standardised or ‘plain’ tobacco packaging was introduced in Australia in December 2012 and is currently being considered in other countries. The primary objective of this systematic review was to locate, assess and synthesise published and grey literature relating to the potential impacts of standardised tobacco packaging as proposed by the guidelines for the international Framework Convention on Tobacco Control: reduced appeal, increased salience and effectiveness of health warnings, and more accurate perceptions of product strength and harm.

**Methods:**

Electronic databases were searched and researchers in the field were contacted to identify studies. Eligible studies were published or unpublished primary research of any design, issued since 1980 and concerning tobacco packaging. Twenty-five quantitative studies reported relevant outcomes and met the inclusion criteria. A narrative synthesis was conducted.

**Results:**

Studies that explored the impact of package design on appeal consistently found that standardised packaging reduced the appeal of cigarettes and smoking, and was associated with perceived lower quality, poorer taste and less desirable smoker identities. Although findings were mixed, standardised packs tended to increase the salience and effectiveness of health warnings in terms of recall, attention, believability and seriousness, with effects being mediated by the warning size, type and position on pack. Pack colour was found to influence perceptions of product harm and strength, with darker coloured standardised packs generally perceived as containing stronger tasting and more harmful cigarettes than fully branded packs; lighter coloured standardised packs suggested weaker and less harmful cigarettes. Findings were largely consistent, irrespective of location and sample.

**Conclusions:**

The evidence strongly suggests that standardised packaging will reduce the appeal of packaging and of smoking in general; that it will go some way to reduce consumer misperceptions regarding product harm based upon package design; and will help make the legally required on-pack health warnings more salient.

## Introduction

Smoking is the largest single cause of avoidable morbidity and mortality in much of the world, being a risk factor for six of the eight leading causes of death globally [1] and responsible for approximately five million deaths a year [2]. Smoking is the risk factor associated with the most deaths per annum in high-income countries and globally only high blood pressure is a greater risk factor [3]. Smoking harms nearly every organ of the body [4], with the adverse health effects of smoking extending beyond the individual smoker, with over 600,000 non-smokers estimated to die each year from exposure to second-hand smoke [5]. Annual public healthcare expenditure in the European Union for treating smoking related illness is estimated to be in excess of 25 billion euros. The European Commission estimates that the life years lost due to smoking related illness corresponds to 517 billion euros a year [6].

In response to these risks the first global public health treaty, the Framework Convention on Tobacco Control (FCTC), was formally initiated at the 48^th^ World Health Assembly in 1995. It came into force in 2005 and is now one of the most widely embraced treaties in the history of the United Nations, with almost 90% of the global population covered through 177 Parties to the Convention, as of August 2013. The objective of the FCTC, as outlined in Article 3, is “to protect present and future generations from the devastating health, social, environmental and economic consequences of tobacco consumption and exposure to tobacco smoke” [7]. To meet this goal the FCTC asserts the importance of both supply issues (e.g. combating illicit tobacco) and also demand reduction measures, including protection from exposure to tobacco smoke, regulation of the contents of tobacco products, cessation, and education, communication, training and public awareness.

Two of these demand reduction measures are controls on tobacco advertising, promotion and sponsorship, and packaging and labelling, identified as priority areas during the development of the FCTC [8], [9]. As a growing number of countries have adopted complete or comprehensive bans on tobacco advertising and promotion, there has been increased regulatory attention paid to the role of packaging as a marketing and communications tool. The guidelines for Articles 11 and 13 of the FCTC recommend Parties introduce plain tobacco packaging [10], [11], which involves standardising pack appearance. In December 2012, the Australian Government became the first to require that all tobacco products be in standardised or ‘plain’ packs. While the Australian High Court ruled in favour of the decision to introduce standardised packaging in Australia in August 2012, a Notice of Arbitration under Australia's Bilateral Investment Treaty with Hong Kong brought by Philip Morris Asia in November 2011, and the World Trade Organization dispute settlement (WT/DS434) brought by Ukraine in March 2012, remain outstanding. Also in December 2012, the European Commission announced the scope of a draft Tobacco Products Directive, which does not provide a pan-European Union mandate for standardised packaging but allows member countries to introduce standardised packaging. Most recently, in February 2013, the New Zealand Government announced, in principle, plans to introduce standardised packaging, as did the Scottish Government in March 2013 and Irish Government in May 2013.

There have been a small number of recent reviews of literature on standardised packaging [12]–[17]. However, none of these reviews adopted a systematic approach and only two were published in peer-reviewed journals.

The primary aim of the systematic review was to assess the impact of standardised tobacco packaging, based upon the potential benefits of standardised packaging proposed by the guidelines for Articles 11 and 13 of the FCTC [10], [11], on: 1) pack and product appeal; 2) prominence of health warnings; 3) use of packaging elements that may mislead about product harm. Secondary aims were to assess any other potential impacts of standardised packaging not identified by the FCTC, assess the facilitators and barriers to plain packaging having an impact, and examine differences in response to standardised packaging, if any, by gender, age, socio-economic status and ethnicity (see the review Protocol [18]). This article reports on the findings for the primary aim of the systematic review and any demographic sub-group differences. The findings for the secondary aims of the review are reported elsewhere (see [19]).

## Methods

The review aimed to include all standardised tobacco packaging primary research studies, conducted since 1980. Twenty-one electronic databases from the fields of health, public health, social science and social care were searched in June and July 2011 as were fourteen websites, including Google Scholar and the Legacy Tobacco Documents Library, a digital archive of tobacco industry documents (see Appendix S1 for a list of the databases and websites, as well as an example of the search strategy). Contact was also made with academics and market research groups known to have conducted research on standardised packaging, either currently or in the past; academics involved in research concerning tobacco packaging, although not specifically standardised packaging; and non-governmental organisations which have written on the topic of standardised packaging; two people known to be collating standardised packaging research within the European Commission and the Australian Department of Health and Ageing respectively. A cut-off date of the 31^st^ August 2011 was set for receipt of full text papers for screening. We did not limit our studies to papers in English, and a number of French studies were included. Studies were managed by the EPPI-Centre's online review software (EPPI-Reviewer 4.0) [20].

A total of 4,518 citations were screened (using the inclusion criteria: from or after 1980; about human populations; about tobacco; about packaging; and primary research) from which 169 papers were retrieved for full text screening by two reviewers. From these, 41 papers were included for data extraction.

### Data extraction

All studies were coded using a standard classification system [21] and further codes were added to capture information specific to this review. A coding tool (see Appendix S2) was developed and data extracted for each study by two researchers, one from the EPPI-Centre (KH/IK) and one from the University of Stirling (KA/RP/SB). Data were extracted on: study aims and design; the sample studied; sampling strategy, recruitment and consent processes; data collection and analysis; and findings (extracted both as a narrative and as odds ratios and standardized mean differences [22]). Authors were contacted for additional information or for clarification if needed.

### Quality appraisal and relevance checking

Different quality criteria were used for each study design, following principles of good practice for critical appraisal of primary research [23], [24]. For surveys, we used a tool developed by Wong et al. [25], and for interventions, we used criteria devised by Shepherd et al. [26]. The relevance of each study was then assessed based on their aims, sample, methods for data collection and analysis and findings. After this stage, two studies were excluded having incomplete analyses, and two excluded on grounds of methodological quality resulting in 37 included studies for the full systematic review [19].

**Figure 1:**
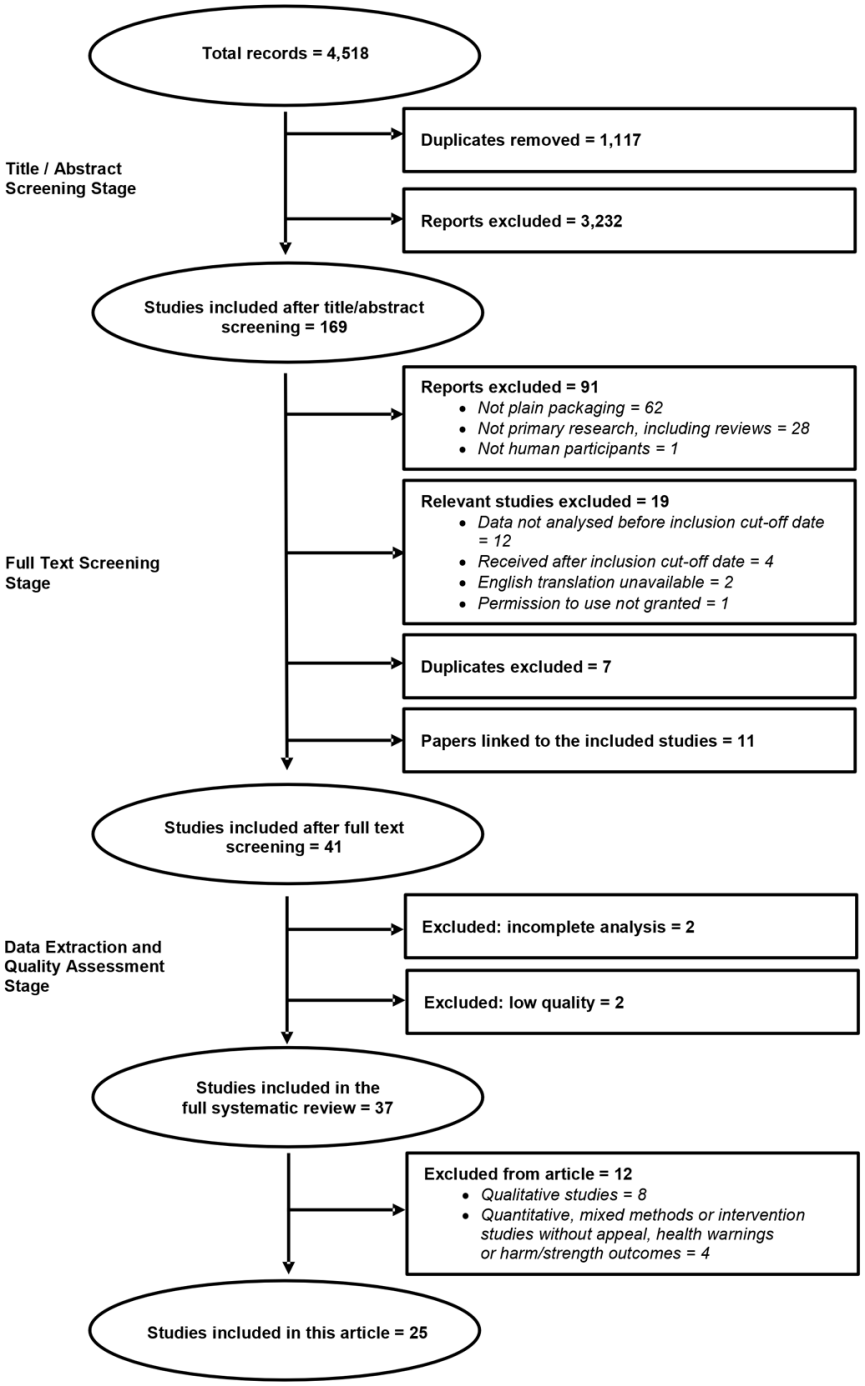
Literature search and study selection process.

This article reports on a sub-set of 25 studies from the full systematic review which report outcomes relating to the potential benefits identified in the guidelines for Articles 11 and 13 of the FCTC, as described above. Eight studies that employed qualitative methods only and four studies that examined other outcomes, such as facilitators and barriers to the introduction of standardised packaging policies or its impact on smoking-related attitudes, beliefs and behavioural intentions, are not included in this paper but their results are outlined elsewhere [19]. We focused on the studies employing quantitative methods only in order to facilitate comparisons and synthesis of results between studies. The literature search and study selection process is shown in Figure 1.

### Synthesis

A framework that encompassed the range of impacts measured was constructed in order to structure the findings [27]. Impacts were organised into overarching themes under which findings are summarised narratively, namely:

- Impact of standardised packaging on appeal
- Impact of standardised packaging on the salience and effectiveness of health warnings
- Impact of standardised packaging on perceptions of product strength and harm.

A narrative synthesis was presented with care taken to avoid ‘vote counting’ of statistically significant results; vote counting fails to take account of the relative size of studies, their methodological quality or the magnitude of their effects [28]. Both statistical significance and directions of effect were examined for each study.

## Results

The 25 quantitative studies reported in this article comprised 18 cross-sectional surveys with an experimental (between- or within-subjects) design, three cross-sectional surveys without an experimental design, three mixed methods studies and one intervention study. Full details and summary findings are given in Table S1.

### Appeal of cigarettes, packs and brands

Twenty-one studies [29]–[49] in the review examined whether and how standardised packs impact on the appeal of cigarettes, packs or brands. The measures of appeal were grouped into three categories, attractiveness of the pack, perceived quality and taste of the cigarettes, and smoker identity – the extent to which the pack was associated with a desirable smoker identity or positive personality attributes. For all 21 studies, Table 1 shows the nature of the comparison made in the study and the direction of effect. ‘Favours branded packs’ means that respondents found the branded packs more attractive than standardised packs or thought that they contained better quality cigarettes or that positive smoker identity attributes were stronger for branded packs than for standardised packs.

**Table 1:** Direction of effect: Attractiveness, quality and smoker identity.

		Direction of effect		
Study	Type of Comparison	Attractiveness	Quality	Smoker Identity
Bansal-Travers 2011 [29]	Branded vs. standardised	Favours branded	Favours branded	
Bondy 1996 [30]	Branded vs. standardised	Favours branded		
Centre for Health Promotion 1993 [31]	Branded vs. standardised	Favours branded	Favours branded	Favours branded
Donovan 1993 [32]	Branded vs. standardised	Favours branded		Favours branded
Doxey 2011 [33]	Branded vs. standardised	Favours branded	Favours branded	Favours branded
Gallopel-Morvan 2010 [34]	Branded vs. standardised	Favours branded	Favours branded	Favours branded
Gallopel-Morvan 2012 [35]	Branded vs. standardised	Favours branded		
Germain 2010 [36]	Branded vs. standardised	Favours branded	Favours branded	Favours branded
Goldberg 1995 [37]	Branded vs. standardised			Favours branded
Hammond 2009 [38]	Branded vs. standardised	Favours branded	Favours branded	
Hammond 2013 [39]	Branded vs. standardised	Favours branded	Favours branded	Favours branded
Hammond 2011 [40]	Branded vs. standardised	Favours branded	Favours branded	Favours branded
Hoek 2009 [41]	Branded vs. standardised	Favours branded		
Hoek 2011 [42]	Branded vs. standardised	Favours branded		
Moodie 2011 [43]	Branded vs. standardised	Favours branded	Favours branded	
Moodie 2012 [44]	Different colours of standardised packs	Standardised rated negatively	Favours lighter-coloured standardised	Standardised rated negatively
Rootman 1995 [45]	Branded vs. standardised	Favours branded		Favours branded
Swanson 1997 [46]	Branded vs. standardised			Favours branded
Thrasher 2011 [47]	Branded vs. standardised	Favours branded		
Wakefield 2008 [48]	Branded vs. standardised	Favours branded	Favours branded	Favours branded
White 2011 [49]	Branded vs. standardised	Favours branded	Favours branded	Favours branded

An empty cell indicates that the study did not address the outcome in question.

#### Attractiveness

Twenty-one studies examined perceptions or ratings of the attractiveness of standardised packs. Findings were highly consistent, with all studies reporting that standardised packs were considered less ‘appealing’, ‘attractive’, ‘cool’, ‘stylish’ and ‘attention-grabbing’ than branded equivalent packs, by both adults and children (see Table 1, third column).

In four studies, three using an experimental between-subjects design [36], [48], [49] and one an experimental within-subjects design [42], comparisons were made of the perceived attractiveness of branded packs against a series of packs retaining progressively fewer original brand elements (brand name font, colour, descriptor terms such as ‘smooth’, and so on). These studies consistently found that packs became less attractive the plainer they became.

In three studies conducted with young women in Canada [33], the UK [39] and the USA [40], an experimental design was used in which current branded female–oriented packs (i.e. where packaging was oriented towards women) were compared in attractiveness with current branded female-oriented packs but with descriptors (terms such as ‘slims’) removed, standardised brown packs for the same female-oriented brands, and current branded packs not oriented towards women. These studies consistently reported that standardised packs were rated as less appealing than branded female-oriented packs, female-oriented packs with descriptors removed, and packs not targeted at women.

Studies conducted with adolescents consistently reported that young people responded negatively to standardised packaging. In a mixed methods non-experimental study with 12–17 year olds in Canada [31], standardised packs were rated significantly (p<0.001) worse on the ratings ugly/attractive, boring/exciting, old-fashioned/modern, awful/nice, dull/colourful and nerdy/cool, while 10–17 year olds in Scotland rated a standardised pack as unattractive (91%), uncool (87%) and a pack you would not like to be seen with (88%) in a non-experimental online survey [44].

#### Perceived quality and taste

The twelve studies which examined perceptions of the quality of cigarettes in standardised packs, using outcomes such as ‘quality of tobacco’, ‘taste’, ‘richness’ and ‘satisfying’, consistently found that cigarettes in standardised packs were perceived as being of lower quality than those in branded packs even when the same brand name appeared on the packs (see Table 1, fourth column). In three experimental studies which compared perceptions of packs with progressively more original branding elements removed [36], [48], [49], ratings of quality became more negative as packs became more standardised. For example, in an experimental between-subjects design study conducted with 14–17 year olds in Australia, ratings of cigarettes as ‘rich’, ‘satisfying’ and ‘high quality’ were lower (p<0.001) for the standardised pack compared with the fully branded pack, and the differences increased as more original branding elements were removed [36]. Similarly, in an experimental between-subjects design study with 16–26 year old female smokers and non-smokers in Brazil, participants rated standardised packs with descriptors as less smooth (p<0.05) and poorer tasting (p<0.001) than branded packs, with the difference in rating increasing as descriptors were removed from the standardised packs [49].

#### Smoker identity

An important aspect of cigarette pack appeal is the extent to which the pack is associated with a desirable smoker identity, and this was examined in thirteen studies. Measures for assessing identity included ratings of packs on projected personality attributes, asking participants whether a pack was aimed at them or someone like them, and visual experiments which measured the strength of association between specific brands and person types. Standardised packs were consistently rated more negatively on desirable personality attributes than branded packs (see Table 1, fifth column). In two experimental between-subjects design studies, 16–26 year old females in Brazil rated standardised packs more negatively than branded packs on the attributes ‘female’, ‘stylish’ and ‘sophisticated’ (p<0.05) [49], while teenagers in Australia rated standardised pack smokers more negatively than branded pack smokers in terms of being ‘young’, ‘masculine’, ‘sociable’ and ‘confident’ [36]. In a visual experiment using a between-subjects design conducted with 14–17 year olds in Australia, respondents' associations between a particular brand and the ‘right’ sort of person (for example, between Marlboro and a rugged outdoor man) weakened or disappeared when the brand was presented in a standardised pack, for four out of six comparisons [46].

#### Subgroup differences

From the studies which examined sub-group differences in the appeal and attractiveness of standardised packs, some patterns emerged. Overall, non-smokers and younger respondents were more affected by standardised packaging. For example, an experimental between-subjects design study with over 1,000 11–49 year olds in Australia found that smokers were significantly *less* likely than non-smokers to rate standardised packaging as ‘unattractive’ (OR = 0.71, 95%CI = 0.52, 0.98), and 11–17 year olds were significantly *more* likely than 18–29 year olds (OR = 2.51, 95%CI = 1.71, 3.68) to rate standardised packs as unattractive [32]. The one study which examined gender differences, an experimental within-subjects design involving 836 French adults, suggested that women found standardised packaging less appealing than men [35], although it was not possible to calculate effect sizes from the information given in the paper. No consistent differences emerged from studies exploring differences in response by ethnicity or socio-economic status.

### Health warnings

Seven studies examined whether standardised packs increase people's ability to notice and recall the health warnings on packs or whether standardised packs affect the perceived seriousness and believability of the warnings [34], [36], [37], [43], [45], [50], [51]. Table 2 illustrates the direction of effect for the results in each of these studies, with ‘favours standardised packs’ meaning that standardised packaging increased the salience and effectiveness of health warnings in terms of recall, attention, believability and seriousness. The overall direction of effect was less consistent than for ‘Appeal’, but overall (four of seven studies) tended to favour standardised packaging.

**Table 2:** Direction of effect: Salience of health warnings.

		Direction of Effect:
Study	Type of Comparison	Salience of Health Warnings (specific measure used)
Beede 1990 [50]	Branded vs. standardised	Favours standardised (recall of warnings)
Gallopel-Morvan 2010 [34]	Branded vs. standardised	Favours standardised (recall of warnings)
Germain 2010 [36]	Branded vs. standardised	No difference (recall of warnings)
Goldberg 1995 [37]	Branded vs. standardised	Multiple analyses reported in 2 papers: mixed results (recall of warnings)
Moodie 2011 [43]	Branded vs. standardised	Favours standardised (noticing, seriousness, believability)
Munafò 2011 [51]	Branded vs. standardised	Favours standardised for non smokers and weekly smokers (attention towards warnings)
Rootman 1995 [45]	Branded vs. standardised	Ontario sample: favours standardised for regular smokers Chicago sample: no difference (recall of warnings, seriousness of warnings)

An experimental between-subjects study that tracked respondents' eye movements (saccades) towards pack images shown on a computer screen suggested that standardised packs attracted more eye movements towards the health warning than did branded packs, among non-smokers (p = 0.001) and weekly smokers (p = 0.001), although there was no difference for daily smokers (p = 0.35) [51]. The impact of health warnings in some studies varied according to the size, type and position of the warnings used. A survey of 12–14 year olds in Canada and the USA reported higher levels of recall of warnings on standardised packs than on branded packs among the Canadian sample but not the American sample [45]. No study examined gender, age or other socio-demographic differences in the effect of standardised packs on response to health warnings.

### Perceptions of harm and strength

Fourteen studies examined whether and how standardised packs impact on perceptions of the harm and strength of cigarette products, packs and brands [29], [33]–[36], [38]–[40], [43], [44], [48], [49], [52], [53]. Three types of outcomes were examined in these studies: perceptions of tar/nicotine levels; perceptions of harmfulness (which includes ratings of which pack, when different types of packs are compared, would be more harmful or risky, would trigger discussions on harmfulness, would inform the smoker about the health effects or would make the smoker think that the cigarettes inside were dangerous); and perceptions of which packs were perceived as ‘easier to quit’. Thirteen of the studies involved comparison between branded and standardised packs, and four of the studies involved comparison between standardised packs which varied in colour and/or the presence or absence of descriptor terms such as ‘smooth’ and ‘gold’.

Table 3 shows the direction of effect for these 14 studies. For perceptions of tar/nicotine levels, ‘favours’ means that the packs were perceived to deliver higher levels of tar/nicotine. For perceptions of harmfulness, ‘favours’ means that the packs were more likely to be associated by respondents with harm or risk. In the final column, ‘favours’ means that the packs were perceived as easier to quit.

**Table 3:** Direction of effect: Perceptions of strength, harmfulness and which packs are easier to quit.

		Direction of Effect		
Study	Type of Comparison	Perceptions of Tar/Nicotine Levels	Perceptions of Harmfulness (specific measure used)	Easier to Quit
Bansal-Travers 2011 [29]	Branded vs. standardised (white)	Favours branded packs	No difference (which buy to reduce health risks)	
Doxey 2011 [33]	Branded vs. standardised (white)	No difference	No difference (health risks compared to other brands)	
Environics Research Group 2008a [52]	Branded vs. standardised (colour not given)		Favours standardised packs (informs about health effects)	
Environics Research Group 2008b [53]	Branded vs. standardised (colour not given)		Favours standardised packs (informs about health effects)	
Gallopel-Morvan 2010 [34]	Branded vs. standardised (brown, grey and white)	Favours branded packs (Branded vs. standardised white and grey).		
		Favours brown standardised pack (standardised brown vs. standardised white and standardised grey)		
Gallopel-Morvan 2012 [35]	Branded vs. standardised (grey)		Favours standardised pack (discussion of and awareness of dangers)	
Germain 2010 [36]	Branded vs. standardised (brown)	No difference (main effect of 3 standardised pack vs. branded packs for 3 different brands; light taste)		
		Favours standardised packs (for 2 out of 3 standardised pack images for one brand comparison only)		
Hammond 2009 [38]	(a) Branded (two different brands) vs. standardised (white)	Favours branded pack	Favours branded pack (3 of 4 comparisons); no difference (1 comparison) (health risks)	Favours standardised pack
	(b) Branded (two different brands) vs. standardised (brown)	No difference (3 of 4 comparisons); favours standardised packs (1 comparison)	No difference (2 of 4 comparisons); favours standardised packs (2 of 4 comparisons) (health risks)	No difference
	(c) Standardised white with descriptor ‘Smooth’ vs. standardised white without descriptor	Favours standardised pack without descriptors	Favours standardised pack without descriptors (health risks)	Favours standardised pack with descriptors
	(d) Standardised brown with descriptor ‘Gold’ vs. standardised brown without descriptor	Favours standardised pack without descriptors	Favours standardised pack without descriptors (health risks)	Favours standardised pack with descriptors
Hammond 2013 [39]	Branded vs. standardised (brown)	Favours standardised packs	Favours standardised packs (health risks)	
Hammond 2011 [40]	Branded vs. standardised (brown)	Favours standardised packs	Favours standardised packs (health risks)	
Moodie 2011 [43]	Branded vs. standardised (brown)		No difference (awareness of health risks)	
Moodie 2012 [44]	Standardised packs of different colours		Favours red standardised packs (level of harm)	
Wakefield 2008 [48]	Branded vs. standardised (brown)	Favours standardised packs		
White 2011 [49]	Branded vs. standardised (brown) with and without descriptors		Favours standardised packs (harmfulness)	Favours packs (branded and standardised) with descriptors
			Favours packs (branded and standardised) without descriptors (harmfulness)	

**Notes to table:** An empty cell indicates that the study did not address the outcome in question.

#### Perceived tar and nicotine levels

Mixed results were reported in the eight studies which measured the impact of standardised packs on perceptions of tar and nicotine strength (Table 3, third column). In some studies, perceptions varied according to the colour of the standardised pack, with darker coloured standardised packs being seen as higher in tar/nicotine, and lighter coloured standardised packs lower, when compared with branded cigarette packs. For example, an experimental within-subjects design survey study conducted with adults in the USA reported that a branded pack was perceived as delivering higher tar than a standardised white pack [29], while in an experimental between-subjects design survey with 15–25 year olds in France, cigarettes in grey and white standardised packs were perceived as lighter strength than in a branded pack (p<0.001), and cigarettes in a brown standardised pack were perceived as stronger than those in grey and white standardised packs (p<0.001) [34]. In two studies involving young women, in the UK and USA, Hammond and colleagues [39], [40] found that brown standardised packs were rated higher in tar than branded packs, suggesting that misperceptions about the relative harmfulness of cigarettes were reduced when darker coloured standardised packs were shown. Conversely, when white standardised packs were compared with branded packs in a survey of young women in Canada, participants perceived no difference between the packs in terms of tar level [33].

#### Perceptions of harmfulness and ease of quitting

Findings from the eleven studies which examined the effect of standardised packs on perceptions of harmfulness and ease of quitting were similarly mixed, although tended to be in the direction of finding standardised packs more effective at conveying impressions of harm or informing about the health effects of smoking (Table 3, fourth and fifth columns). Again the colour of the pack seemed to be important, with, for example, a red standardised pack being perceived as more harmful than a green, white or blue standardised pack in a survey of 10–17 year olds in Scotland [44].

An online experimental cross-sectional survey with 516 adult smokers and 806 youth in the UK included ratings of which pack was perceived to have the most tar, which would reduce the risks to health, and which would be easier to quit (asked of the adult sample), across four paired comparisons: branded vs. standardised white packs (for two different brands), branded vs. standardised brown packs (for two different brands), standardised white packs with and without a descriptor term ‘Smooth’, and standardised brown packs with and without a descriptor term ‘Gold’ [38]. There were differences in perceptions of standardised packs depending on the colour. Branded packs were perceived as more harmful than standardised white packs, but standardised brown packs were perceived as equally harmful or more harmful than branded packs. While standardised white packs were perceived as easier to quit than branded white packs, standardised brown packs were perceived as no easier to quit than branded brown packs. These findings suggest that brown is a more effective colour than white for standardised packs, as white is generally associated with lesser harm. The addition of descriptor terms ‘Smooth’ (on white standardised packs) and ‘Gold’ (on brown standardised packs) had the effect of making the standardised pack with descriptors appear to be lower in health risk and easier to quit (p<0.001 for all ratings) than the standardised pack without descriptors. This suggests that even on standardised packs, the addition of descriptor terms can mislead smokers about the harmfulness of the product.

#### Subgroup differences

Studies which compared sub-group differences in participants' responses found that in general, smokers were more likely to have misperceptions about the harmfulness of packs, both standardised and branded, than non-smokers. For example, a survey of 16–19 year olds in the UK found that smokers were more likely than non-smokers to believe that both branded and standardised packs would be a lower health risk (β = 0.08, p<0.027) and contain less tar (β = 0.13, p = 0.001) [39]. Few direct comparisons were made in respect to age, gender or other socio-demographic differences, and no consistent pattern emerged from these.

## Discussion

### Main findings

This review examined 25 quantitative studies that explored consumer perceptions or responses to the impact of standardised or ‘plain’ packaging of tobacco products on appeal, salience and effectiveness of on-pack health warnings, and perceptions of product strength and harm. As the review was carried out when no country had introduced standardised packaging, studies were limited to experimental designs, surveys or observational studies. Despite the range of designs, there was considerable consistency between study findings. Overall, the available research suggested consistently that standardised or plain packaging reduced the appeal of cigarettes. Although findings were mixed, standardised packs also tended to increase the salience of health warnings and to address smokers' misconceptions about product strength and harm that arise from existing branded packs.

### Wider applicability

Findings from the review are consistent in a number of respects with the wider marketing literature, where packaging is a well-established marketing tool [54], [55]. Investment in packaging is seen as important [56] and to be successful packaging must appeal visually and create a positive impression [57], [58]. The UK and other tobacco markets have seen extensive cigarette pack innovations in recent years [59]–[61]. The tobacco industry has explicitly highlighted the positive effects that innovative packaging can have on sales [62], [63].

The findings in respect to pack colour are also consistent with wider literature. For a wide range of consumer goods, pack colour is considered one of the most important features of packaging design [55], [56] as it can heighten pack appeal and influence product perceptions and choice [64]–[66]. It is also well established that pack colour can be used for tobacco products to communicate product strength and harm [67], [68], as indicated in the tobacco industry's own internal documents [69]–[72]. This is misleading, as all conventional cigarettes pose a similar health risk, given that smokers can alter the way they smoke cigarettes of different tar and/or nicotine machine-measured yields in order to compensate for differences and satisfy their nicotine addiction [73]. In addition there is no evidence that brands differ in ease of quitting. The pack is often viewed as central to product evaluation [74] and the findings indicate that many consumers equate pack colour with product strength, tar delivery, health risk and harm. This misunderstanding has important implications for consumer protection.

There are also similarities between findings in the included studies on health warnings, and the wider warnings literature. Globally, on-pack health warnings vary considerably, particularly in respect to type, size and positioning [75]. This may reflect uncertainty about best practice given that the FCTC only published detailed guidelines on Article 11 at the end of 2008 [10]. However, that larger pictorial warnings, prominently displayed on the pack front rather than on reverse or side panel, appeared most salient is consistent with the literature [76].

### Limitations

This review had a number of limitations. As standardised packaging research can never truly replicate real market conditions until it is fully implemented, as it was in Australia in December 2012, a full evaluation of the real world impact was not possible at the time of the review. This limits the types of study design that can be employed to assess standardised packaging, with designs which help increase confidence in the findings (such as randomised control trials or before-and-after designs) not feasible [77]. Another limitation is the use of convenience or probability samples, which limits sample representativeness. In addition, all studies looked at cigarettes and excluded other tobacco products. Likewise, all come from a small number of high-income countries in Australasia, North America or Western Europe. This is informative as it is these countries that are most likely to introduce such a policy, but provides no insight into the potential impact of standardised packaging in developing nations, although the pattern of findings across different population sub-groups provides some expectation that findings might be applicable across countries; however, more research is needed to determine whether this might be the case. The potential impact of standardised packaging in developing countries is a concern as the number of annual deaths related to tobacco use is expected to rise to eight million within the next two decades, with 80% of these deaths projected in low- and middle-income countries [78].

In addition, the included studies also failed to consider level of nicotine dependence among smokers and analysis seldom considers ethnicity or socio-economic status, which limits our understanding of possible impacts upon different population segments. In this particular article, studies employing qualitative methods were not included, but they were reported in the wider review that served as the basis for this work, and these qualitative studies contained similar themes and findings [19].

### Strengths

This review differs from previous reviews [12]–[17] in that it employs a systematic approach, where included studies are identified following careful and extensive searches. While this does not ensure that all relevant studies have been captured, at least until the cut-off date, it does provide confidence that best practice with regard to searching has been followed. This, and the fact that the included studies were checked for relevance and methodological rigour, can be considered strengths of this systematic review. In addition, the review methods took account of the fact that some of the authors had been involved in conducting individual studies that met the inclusion criteria for the review. This type of perceived conflict of interest can arise in systematic reviews conducted in specialised research areas. To minimise the risk of bias, no member of the research team who had been previously involved in packaging research extracted data, assessed study relevance or quality, or decided upon study inclusion.

Finally, while there were a limited number of studies and designs within the review, the findings were largely consistent across different designs, countries, populations, smokers and non-smokers suggesting that we can be fairly confident about the potential effects of standardised packaging.

### Future research

The extant literature strongly suggests that standardised packaging will reduce pack, product and user appeal, that it will go some way to reduce consumer misperceptions regarding product harm based upon package design, and will help make the legally required on-pack health warnings more salient. Further research can build upon the existing findings in a number of ways. Research in low- and middle-income countries, ideally exploring perceptions of standardised packaging for a range of tobacco products, would be informative. Research using study designs that more closely approximate what consumers experience while using standardised packs in naturalistic settings could help provide insight into the potential impacts of standardised packaging, at least in countries where is has not been introduced. In Australia, with all legitimate tobacco products on the market available only in standardised packs, research exploring the impact of standardised packaging on consumer cognitions, emotions and behaviours is required. The use of longitudinal research, pre- and at multiple time-points post-standardised packaging, to monitor the perceptions of youth and adult smokers and non-smokers would be of considerable value. Such research, and indeed studies elsewhere, should build income, ethnicity and dependence level, where possible, into the sampling strategy.

## Supporting Information

Appendix S1 **Databases & Search Strategy.**
(DOCX)

Appendix S2 **Data Extraction Coding Tool.**
(DOCX)

Table S1 **Methodological characteristics and main findings of quantitative studies examining appeal, salience of health warnings and perceptions of product harm/strength.**
(DOCX)

Checklist S1 **PRISMA Checklist.**
(DOCX)
