# Supplementary material for: Is Consumer Response to Plain/Standardised Tobacco Packaging Consistent with Framework Convention on Tobacco Control Guidelines? A Systematic Review of Quantitative Studies
Source: PLoS One. 2013 Oct 16;8(10):e75919. doi: 10.1371/journal.pone.0075919 (PMC3797796; doi:10.1371/journal.pone.0075919)
Supplement: Appendix S1 — Databases & Search Strategy. (DOCX) [file pone.0075919.s001.docx]

**APPENDIX S1: DATABASES & SEARCH STRATEGY**

**Electronic Databases**

- ABI INFORMGlobal and ABI/INFORM Archive Complete
- ASSIA (Applied Social Sciences Index and Abstracts)
- Business Source Premier
- CAB Abstracts
- Cochrane Library
- Conference Papers Index
- Conference Proceedings Citation Index- Social Science & Humanities
- EconLit
- EMBASE
- ERIC (Education Resources Information Center)
- FRANCIS
- Health Promis
- HMIC (Health Management Information Consortium)
- IBSS (International Bibliography of the Social Sciences)
- Index to Theses (UK and Ireland)
- Proquest Dissertation and Theses
- PsycINFO
- PubMed
- Social Policy and Practice
- Social Policy Digest
- Social Science Citation Index
- Sociological Abstracts
- TROPHI (Trials Register of Public Health Interventions)
- Zetoc

**Other catalogues and websites:**

- Advertising Education Forum database
- CDC Smoking and Health Resource Library
- Dart Europe (theses)
- ECDC: European Centre for Disease Prevention and Control
- Google Scholar
- King’s Fund Library
- Legacy Tobacco Documents Library (includes the British American Tobacco Archive)
- OpenGrey (System for information on Grey Literature in Europe)
- Social Science Research Network
- UK Tobacco Industry Advertising Documents Database
- WHO: World Health Organization
- World Advertising Research Center
- WorldCat Library Catalogue
- A database of studies collected for a previous EPPI-Centre review on young people’s access to tobacco.

**Example search: PubMed 23^rd^ June 2011, yielding 687 records**

((cigar*[tiab] OR hand-roll*[tiab] OR HRT[tiab] OR make-your-own[tiab] OR MYO[tiab] OR roll-your-own[tiab] OR RYO[tiab] OR smok*[tiab] OR tobacco[tiab] OR kretek[tiab] OR bidis[tiab] OR beedis[tiab] OR snus[tiab] OR snuff[tiab] OR chew*[tiab] OR gutk*[tiab] OR zarda[tiab] OR pan mas*[tiab] OR paan[tiab] OR betel[tiab] OR beedi[tiab] OR bidi[tiab] OR rollie*[tiab]) OR (smoking[mh:noexp] OR tobacco use cessation[mh] OR tobacco industry[mh])) AND ((pack design[tiab] OR pack designs[tiab] OR packet design[tiab] OR packet designs[tiab] OR package design[tiab] OR package designs[tiab] AND (("1980"[PDat] : "2011"[PDat]))) OR (product packaging[mh:noexp] OR product labeling[mh]) OR ((((descriptor[tiab] OR descriptors[tiab] OR branding[tiab] OR brand[tiab] OR brands[tiab] OR trade mark[tiab] OR trade marks[tiab] OR trade marking[tiab] OR graphics[tiab] OR graphical[tiab] OR graphic[tiab] OR logo[tiab] OR logos[tiab] OR vignette[tiab] OR vignettes[tiab] OR liveried[tiab] OR image[tiab] OR images[tiab] OR design[tiab] OR designs[tiab]) AND (remove[tiab] OR removal[tiab] OR removing[tiab] OR absence[tiab] OR restricted[tiab] OR restriction[tiab] OR restrictions[tiab] OR restrict[tiab] OR outlaw[tiab] OR outlawing[tiab] OR ban[tiab] OR bans[tiab] OR prohibition[tiab] OR prohibit[tiab])) OR (dissuasive[tiab] OR generic[tiab] OR homogenous[tiab] OR plain[tiab] OR plainer[tiab] OR plainest[tiab] OR standard[tiab] OR standardised[tiab] OR standardized[tiab] OR unbranded[tiab] OR no-frills[tiab] OR unliveried[tiab] OR neutral[tiab] OR shape[tiab] OR shapes[tiab])) AND (pack[tiab] OR packet[tiab] OR package[tiab] OR packs[tiab] OR packaging[tiab] OR packets[tiab] OR packages[tiab] OR pouch[tiab] OR pouches[tiab] OR tin[tiab] OR tins[tiab] OR container[tiab] OR containers[tiab] OR carton[tiab] OR cartons[tiab] OR softpack[tiab] OR softpacks[tiab] OR hardpack[tiab] OR hardpacks[tiab] OR canister[tiab] OR canisters[tiab] OR plastic can[tiab] OR cans[tiab] OR cardboard can[tiab])) AND (("1980"[PDat] : "2011"[PDat])))
