# Supplementary material for: Is Consumer Response to Plain/Standardised Tobacco Packaging Consistent with Framework Convention on Tobacco Control Guidelines? A Systematic Review of Quantitative Studies
Source: PLoS One. 2013 Oct 16;8(10):e75919. doi: 10.1371/journal.pone.0075919 (PMC3797796; doi:10.1371/journal.pone.0075919)
Supplement: Appendix S2 — Data Extraction Coding Tool. (DOCX) [file pone.0075919.s002.docx]

**APPENDIX S2: DATA EXTRACTION CODING TOOL**

- **Study Aims and Design**
  - What are the broad aims of the study?
    *Write in authors' description*
    - Explicitly stated (give details)
      - Info
    - Implicit
    - Unclear / not stated
  - Which impacts are measured?
    - Salience of health warnings
      *e.g. prominence/seriousness/visibility/comprehension/understanding/believability*
    - Attractiveness of packaging and the product
      *e.g. appeal/desirability/value/judgements*
    - Perceptions of product strength and harm
      *e.g. understanding/awareness/judgements*
    - Smoking related intentions
    - Attitudes, beliefs or feelings towards smoking
    - Pack preferences
      *e.g. preference/liking for plain packs that differ by shape or method of opening*
    - Knowledge of tobacco harms, constituents, ingredients
    - Knowledge, attitudes, beliefs or feelings towards the brand
    - Attitudes, beliefs or feelings towards the packaging or product
    - Support for plain packaging
      *e.g. Public approval ratings*
    - others (please specify)
  - What outcomes from plain packaging were identified by the authors?
    - Preventing smoking initiation
    - Preventing smoking initiation for young people
    - Aiding smoking cessation
    - Reducing consumption
    - Other (give details)
    - None identified
  - What is the focus of the research?
    - Individual level effects of plain packaging
    - Views on the introduction of plain packaging policies
    - Both
    - Other
  - How was the study funded?
    - Explicitly stated
    - Implicit
    - Unclear/not stated
  - Study design
    - Intervention Study
      - RCT
      - Controlled trial (not randomised)
      - Correlational / observational study
      - Pre and post test
      - Post test only
      - Other design
    - Surveys
      - Cross sectional
      - Longitudinal
    - Qualitative
  - Country (give details)
    - UK
    - Other Europe
    - North America
    - South America
    - Australasia
    - Asia
    - Africa
  - Date of publication
    - Please state
  - Type of publication
    - Journal (peer reviewed)
    - Journal
    - Report
    - Newspaper / magazine article
    - Thesis
    - Other
  - Quality of reporting
    - Yes, good quality
      *Use if aims explicitly stated*
    - Yes, clear to some extent
    - No - insufficient detail
  - Relevance of the aims for the plain packaging review
    - Aims solely focused on plain packaging
    - Aims include some explicit focus on plain packaging
    - Aims implicitly suggest some focus on plain packaging
- **Actual Sample**
  - What was the total number of participants in the study?
    - Explicitly stated
    - Implicit
    - Unclear / not stated
  - What are the ages of the participants?
    - Give details
    - Not stated
  - What is the gender of the participants?
    - Female
    - Male
    - Mixed gender (numbers for each)
  - What is the SES of participants?
    - Explicitly stated
    - Implicit
    - Unclear/not stated
  - What is the ethnicity of participants?
    - Explicitly stated
    - Implicit
    - Unclear / not stated
  - What is the smoking status of participants?
    - Smokers only
    - Non-smokers only
    - Mixed smokers / non smokers
    - Unclear / not stated
  - Other key characteristics not captured above
    - Details
  - Quality of reporting
    - Yes, good quality
      - yes
    - insufficient detail
  - Relevance of sample for review on plain packaging
    *Guidance: Plain packaging would seem to be most relevant for non-smoking youth and smokers of all ages. As UK industry marketing documents often highlight the importance of young adults (18-35) given that they are considered to be more brand conscious, perhaps there is one group that is a less relevant sample when it comes to plain packaging, that of non-smoking older adults aged 36 years and above - especially as very few older adult non-smokers take up the habit. If sample includes non smokers over 36, then it should be considered a sample of moderate relevance.*
    - Highly relevant
    - Moderate relevance
    - Limited relevance
- **Sampling, recruitment and consent**
  - What was the sampling strategy?
    - Explicitly stated
    - Implicit
    - Unclear / not stated
  - What was the setting for the recruitment of the sample?
    - Details
  - What population is the sample attempting to represent?
    - Nationally representative sample
    - Regionally representative sample
    - Purposive sample
    - Convenience sample
    - Other
    - Unclear
  - Are the level of the authors conclusions appropriate given the population?
    - Yes, appropriate level conclusions
    - No inappropriate level conclusions
  - How were people recruited to take part in the study?
    - Explicitly stated
    - Implicit
    - Unclear/not stated
  - Was consent sought?
    - Participant consent sought
    - Other consent sought
    - Consent not sought
    - Unclear / not stated
  - Was information provided before participants agreed to participate?
    - Information provided to participants
    - Information provided to others
    - Unclear / not stated
  - Was the study approved by an ethics committee?
    - Yes
    - Unclear / not stated
  - Attempts to preserve confidentiality/anonymity of the respondents?
    - Explicitly stated
    - Implicit
    - Unclear / not stated
  - Quality of reporting - sampling and recruitment
    - yes, sampling and recruitment clearly reported
    - To some extent
    - No
  - Is the study ethically robust?
    - Yes
    - To some extent
    - No
- **For surveys/views on packaging designs/images**
  - description of plain pack (give details)
  - description of comparison packs (give details)
- **Data Collection and analysis**
  - What method was used to collect data?
    - Focus group interview
    - One to one interview
    - Observation
    - Self completion questionnaire
    - Self completion report or diary
    - Hypothetical scenario including vignettes
    - Online survey
    - Other
    - Not stated
  - What types of questions were asked?
    - Closed questions
    - Open ended questions
    - Both types
    - Not stated
  - Who collected the data?
    - Explicitly stated
    - Implicit
    - Unclear
    - Not stated
  - Do the authors describe data analysis methods?
    - Yes
    - Not specified
    - Unclear
  - Do the authors provide a rationale for the methods used for data analysis?
    - Yes
    - No
  - Quality reporting - collection/analysis
    - Yes
    - Partially
    - No
  - Relevance - collection/analysis
    - Yes
    - To some degree
    - No
    - Unclear
- **Findings**
  - What is the extent of findings on plain packaging?
    - Findings exclusively on plain packaging
    - Broader findings but with major focus on plain packaging
    - Broader findings with limited focus on plain packaging
  - For studies that focus on the individual level effects
    - What was the effect of plain packaging on knowledge?
      - Give details
      - No effect reported
      - Unclear
    - What the reason for the effect on knowledge?
      - Salience of health warnings
      - Appeal of the package
      - Perceptions of harm
      - Other
    - What was the effect of plain packaging on attitudes and/or beliefs?
      - Give details
      - No effect reported
      - Unclear
    - What was the reason for the effect on attitudes and /or beliefs?
      - Salience of health warnings
      - Appeal of the package
      - Perceptions of harm
      - Other
    - What was the effect of plain packaging on behaviour?
      - Give details
      - No effect reported
      - Unclear
    - What was the reason for the effect on behaviour?
      - Salience of health warnings
      - Appeal of package
      - Perceptions of harm
      - Other
    - Associations between plain packaging and which other variables are reported
      - None
      - Gender
        - Significant (give details)
        - Not significant
      - Age
        - Significant (give details)
        - Not significant
      - Ethnicity
        - Significant (give details)
        - Not significant
      - Smoking status
        - Significant (give details)
        - Not significant
      - Socioeconomic status
        - Significant
        - Not significant
      - Other tobacco control policies
        - Significant (give details)
        - Not significant
      - Other
  - For studies on views about the introduction of plain packaging policies
    - What data are available on views on plain packaging policies?
      - Give details
      - None
    - List any benefits or harm identified to the introduction of plain packaging
      - Benefits
      - harm
      - None reported
    - Do the results highlight any facilitators or barriers to plain packaging? Tick all that apply
      - Facilitators
        - Policymaker understanding of plain packaging - supportive
          *Acceptance of packaging as a promotional tool ii) Recognising that plain packaging may, on its own, or part of a comprehensive suite of measures, have potential public health benefits*
        - Related policy and intervention supporting the use of plain packaging
          *A ban on all other forms of marketing ii) Strong tobacco control policies in general*
        - Public and political support for plain packaging
          *i) High acceptance of plain packaging as a potential policy measure by governments ii) Approval of plain packaging from the public (both smokers and non-smokers)*
        - Environmental considerations
          *i) Wide ranging marketing restrictions – absence of tobacco marketing the norm ii) Declining smoking prevalence iii) High percentage of smokers wanting to quit*
      - Barriers
        - Policymaker understanding of plain packaging – not supportive
          *i) Packaging not accepted as a promotional tool ii) Not recognising that plain packaging, whether on its own or as part of a comprehensive suite of measures, has potential public health benefits*
        - Related policy and intervention not supporting the use of plain packaging
          *i) Use of large health warnings or those with pictorial images ii) Ban of displays of tobacco within the retail environment ii) Weak tobacco control policies in general*
        - Lack of public and political support for plain packaging
          *Low acceptance of plain packaging as a viable policy measure by governments ii) Lack of approval of plain packaging from the public (both smokers and non-smokers)*
        - Environmental considerations
          *i) The possible impact on the illicit tobacco trade, and price of tobacco ii) Difficult financial climate iii) Potential of increasing smoking uptake*
        - Tobacco industry and retailer response
          *i) Tobacco industry opposition; threats of legal action, reduced pricing by tobacco companies, potential impact on stimulating illicit trade and resultant loss of government taxes, etc ii) Retail industry opposition; impact on serving time, additional staff costs, inability to distinguish genuine from illicit product*
        - Physiological
          *Nicotine addiction*
      - Other barriers and facilitators identified
      - None reported
  - Other findings
    - Give details
  - Relevance of the findings
    - Yes
    - To some degree
    - No
    - Unclear
