# Supplementary material for: Is Consumer Response to Plain/Standardised Tobacco Packaging Consistent with Framework Convention on Tobacco Control Guidelines? A Systematic Review of Quantitative Studies
Source: PLoS One. 2013 Oct 16;8(10):e75919. doi: 10.1371/journal.pone.0075919 (PMC3797796; doi:10.1371/journal.pone.0075919)
Supplement: Table S1 — Methodological characteristics and main findings of quantitative studies examining appeal, salience of health warnings and perceptions of product harm/strength. (DOCX) [file pone.0075919.s003.docx]

**Table S1: Methodological characteristics and main findings of quantitative studies examining appeal, salience of health warnings and perceptions of product harm/strength**

| **Study**  **Relevance & Quality Rating** | **Population** | **Aim**  **Design & Data collection methods** | **Description of standardised pack**  **Nature of comparison: branded pack and standardised pack** | **Findings** |
| --- | --- | --- | --- | --- |
| **Quantitative studies (*n* = 21)** | | | | |
| ***Cross-sectional surveys with an experimental (between- or within-subjects) design (*n *= 18)*** | | | | |
| **Bansal-Travers 2011**  **Rel:** Medium  **Qual:** Medium | **Location:** USA  **Sample size:** 397  **Age:** 18-55+ years  **Gender:** mixed  **Smoking status:** mixed  **Sample type:** convenience sample | To examine the impact of cigarette pack design, product descriptors, and health warnings on risk perception and brand appeal  Survey (with experimental within-subjects design)  Interview method | Standardised white, name of brand in standardised text and number of cigarettes in box. No health warning or descriptors.  Twelve fictitious packs rated in pairs, only one of which included a standardised pack compared with a branded pack. | **Appeal: Attractiveness** Branded pack rated (p<0.001) more attractive and more likely to be bought than standardised pack.  **Appeal: Quality** Branded pack rated (p<0.001) more smooth tasting and better quality than standardised pack.  **Harm and Strength** Participants asked which pack they would expect to deliver the most tar and which they would buy if trying to reduce the risks to health. Branded pack rated (p<0.001) more likely than standardised pack to deliver most tar; no difference between selecting standardised or branded pack to reduce health risks.  **Harm and Strength: Sub-Groups** No significant differences between smokers and non-smokers. Results remained consistent after logistic regression analyses were conducted, adjusting for age, gender, race/ethnicity, and education. |
| **Donovan 1993**  **Rel:** High  **Qual:** Medium | **Location:** Australia  **Sample size:** 1070  **Age:** 11-49 years  **Gender:** mixed  **Smoking status:** mixed  **Sample type:** convenience sample | To assess the appeal of standard (standardised) packs  Survey (with experimental between-subjects design)  Interview method | Standard (standardised) packs with text health warnings and in red and blue colour, but in lighter shades. Colours alternated across respondents.  Random allocation to 1 of 3 conditions: branded packs; branded packs with new text health warnings; standard (standardised) packs. After attribute ratings, respondents in both the current branded packs conditions were shown the standard packs. | **Appeal: Attractiveness** Standardised packs rated less attractive, less colourful and more likely to deter purchase than branded packs (significance values not given).  **Appeal: Smoker Identity** Standardised packs perceived by young smokers and non-smokers as more appropriate to *‘old people’* than *‘young people*’, and less *‘ok to be seen with’* (significance values not reported) compared with branded packs.  **Appeal: Sub-Groups** Smokers significantly less likely than non-smokers to rate standardised packaging ‘unattractive’ (*OR* = 0.71, 95%CI = 0.52, 0.98)*. Smoking and non-smoking 11-17 year olds were significantly more likely than 18-29 year olds in the study to rate standardised packaging ‘unattractive’ (*OR* = 2.51, 95%CI = 1.71, 3.68)*. |
| **Doxey 2011**  **Rel:** Medium  **Qual:** Medium | **Location:** Canada  **Sample size:** 512  **Age:** 18-25 years  **Gender:** females only  **Smoking status:** mixed  **Sample type:** convenience sample | To examine the impact of female-oriented cigarette packaging and standardised packaging on women’s perceived appeal, taste, tar yield, health risks and smoker traits  Survey (with experimental between-subjects design)  Online | Standardised packs (condition 3) were of white background colour, generic black font (brand name and number of cigarettes) and the same shape/size of container as the original.  8 packs, viewed one at a time, in 4 conditions: (1) female-oriented; (2) female-oriented without descriptors; (3) female-oriented without descriptors or brand imagery, standardised with a white background; (4) leading Canadian cigarette brands with no overtly feminine characteristics. All packs displayed same pictorial health warning covering 50% of principal display surface. | **Appeal: Attractiveness** Standardised packs rated (p<0.05) less appealing than branded packs aimed at women for all except 3 brands (Camel, XS, Silk Cut). Standardised packs given lower appeal ratings than packs targeted at men (p=0.004). Linear regression: branded packs rated more appealing than packs with no descriptors (p=0.02), packs targeted at men (p<0.001) and standardised packs (p<0.001).  **Appeal: Quality** Standardised packs given lower taste rating (p<0.05) than 3 branded packs with flavour descriptors (Benson and Hedges Menthol, Capri Vanilla, Capri Cherry). Linear regression: branded packs higher taste ratings than standardised packs (p<0.001), packs with no descriptors (p=0.01), and packs targeted at men (p=0.004). Standardised packs rated lower in taste than packs targeted at men (p=0.01) and packs with no descriptors (p=0.004)  **Appeal: Smoker Identity** Examined strength of ‘smoker trait’ associations (‘*female’, ‘slim’, ‘glamorous’, ‘cool’, ‘popular’, ‘attractive’* and ‘*sophisticated’*, ‘*exciting’)*  with different cigarette packs. Branded packs given higher (p<0.001) trait scores than standardised packs.  **Harm and Strength** Few differences across conditions in perceived tar yield and health risk ratings for individual packs. No significant main effects were observed for standardised packs. |
| **Environics Research Group 2008a**  **Rel:** Medium  **Qual:** Medium | **Location:** Canada  **Sample size:** 1000  **Age:** 12-18 years  **Gender:** mixed  **Smoking status:** mixed  **Sample type:** national representative sample | To examine the effect of different sizes of health warning messages on branded and standardised packs among youths  Survey (with experimental between-subjects design)  Interview method | No details of colour but standardised packs had a brand name and health warning messages but no brand markings such as colours, fonts and logos.  6 different mock-up packs of a possible 24: 16 branded packs (2 health warning messages in 4 sizes for 2 brands) and 8 standardised packs (2 health warning messages in 2 sizes for 2 brands). | **Harm and Strength** Standardised packs rated more likely than branded packs to inform about health effects.  Warning size = 50% pack face: Branded 25%, Standardised 50%, Both equally effective 21%, Neither 3%.  Warning size = 75% pack face: Branded 26%, Standardised 52%, Both equally effective 19%, Neither 3%.  **Harm and Strength: Sub-Groups** Smokers more likely than non-smokers to rate both standardised and branded packs with 75% health warnings equally effective at informing about health effects of tobacco (25% vs. 19%); non-smokers more likely than smokers to rate branded packs with 50% health warnings more effective (26% vs. 20%).  In standardised pack comparison with 50% health warning, 15-18 yr olds more likely than 12-14 yr olds to rate standardised pack more effective (53% vs. 46%).  Girls more likely than boys to rate branded pack with 75% size health warning most effective (29% vs. 22%). |
| **Environics Research Group 2008b**  **Rel:** Medium  **Qual:** Medium | **Location:** Canada  **Sample size:** 1000  **Age:** 18-55+  **Gender:** mixed  **Smoking status:** smokers only  **Sample type:** national representative sample | To examine the effect of different sizes of health warning messages on branded and standardised packs among adults  Survey (with experimental between-subjects design)  Interview method | No details of colour but standardised packs had a brand name and health warning messages but no brand markings such as colours, fonts and logos.  6 different mock-up packs of a possible 24: 16 branded packs (2 health warning messages in 4 sizes for 2 brands) and 8 standardised packs (2 health warning messages in 2 sizes for 2 brands). | **Harm and Strength** Standardised packs rated more likely than branded packs to inform about health effects.  Warning size = 50% pack face: Branded 20%, Standardised 48%, Both equally effective 25%, Neither 7%.  Warning size = 75% pack face: Branded 19%, Standardised 50%, Both equally effective 25%, Neither 6%.  **Harm and Strength: Sub-Groups** No significant variations in rating which pack more effective in informing about health effects of smoking among key demographic and attitudinal groups, except: university graduates (57%) more likely than those who had not finished high school (43%) to choose standardised pack with 75% warning. |
| **Gallopel-Morvan 2010**  **Rel:** High  **Qual:** Medium | **Location:** France  **Sample size:** 540  **Age:** 15-25 years  **Gender:** mixed  **Smoking status:** mixed  **Sample type:** convenience sample | To assess the appeal and prominence of health warnings on branded packs and grey, white and brown standardised packs  Survey (with experimental between-subjects design)  Interview method  Hypothetical scenario | Grey, white or brown standardised pack, with name of the brand, same font, same size and the usual health warning *Fumer tue* covering 30% of the front of the pack.  Viewed a picture of either the branded pack of the leading cigarette brand in France or 1 of 3 standardised packs (grey, white or brown). | **Appeal: Attractiveness** Branded pack rated higher (p<0.001) than three standardised packs (grey, white and brown) as ‘attention grabbing’, ‘attractive’, ‘nice’ and ‘flashy’.  **Appeal: Quality** Branded pack rated statistically significantly higher (p<0.001) than three standardised packs (grey, white and brown) as ‘expensive’ and containing ‘good quality’.  **Appeal: Smoker Identity** Branded pack rated higher (p=0.018) than grey standardised pack in terms of ‘targeting young people’.  **Health Warnings** Health warning rated more prominent on standardised packs than on branded pack (p<0.001), and brand name rated less prominent on standardised packs than on branded pack (p=0.023). No significant differences were found across the three standardised packs (grey, white and brown) in respect to the prominence of the warning or brand name.  **Harm and Strength** Grey and white standardised packs perceived to contain ‘lighter’ cigarettes than branded pack and brown pack (p<0.001) (in the study, ‘lighter’ appeared to combine both lower tar and lighter tasting). |
| **Gallopel-Morvan 2012^§^**  **Rel:** High  **Qual:** Medium | **Location:** France  **Sample size:** 836  **Age:** 18-65+  **Gender:** mixed  **Smoking status:** mixed  **Sample type:** national representative sample | To examine perceptions of regular packs, limited edition packs and standardised versions of the same cigarette brands (a) or regular packs and standardised versions of the same cigarette brands (b)  Survey (with experimental within-subjects design)  Interview method  Hypothetical scenario | Grey with brand name in black standardised font and text warning *Fumer tue* on pack front  Viewed three cigarette packs for the same brand (from 1 of 3 leading brands in France): a regular, a limited edition and a standardised pack. No specific features compared. | **Appeal: Attractiveness** Standardised packs rated (p<0.001) less likely to attract attention and less attractive than branded packs.  **Appeal: Sub-Groups** Slightly more smokers than non-smokers rated standardised pack as ‘disgusting’ rather than ‘attractive’ but difference was not statistically significant (*OR* = 1.18, 95%CI = 0.89, 1.58)*. Fewer smokers than non-smokers rated the standardised pack as ‘does not motivate to buy’, but difference not statistically significant (*OR* = 0.79, 95%CI = 0.58, 1.07)*. Under 25 year-olds more likely than older respondents, and men more likely than women, to rate a branded pack when compared with a standardised pack as containing ‘cigarettes of good quality’ (p<0.05). Women more likely than men to rate standardised packs as ‘disgusting’ rather than ‘attractive’ (p<0.01). Not possible to calculate effect sizes from the information given in the paper.  **Harm and Strength** Standardised pack perceived as more likely (p<0.01) than current branded pack to trigger discussions on the dangers of tobacco and make people think that the cigarettes inside the pack are dangerous.  **Harm and Strength: Sub-Groups** Non-smokers more likely than smokers to choose standardised pack as the most effective to trigger discussions on the dangers of smoking (p<0.01) and make people think that cigarettes inside the pack are dangerous (p<0.05). No significant differences by age or gender. |
| **Germain 2010**  **Rel:** High  **Qual:** Medium | **Location:** Australia  **Sample size:** 1087  **Age:** 14-17 years  **Gender:** mixed  **Smoking status:** mixed  **Sample type:** convenience sample | To examine the effect of progressively more ‘standardised’ packaging on adolescents’ perceptions of pack appeal and cigarette taste, plus recall of health warnings  Survey (with experimental between-subjects design)  Online | Four standardised cardboard brown packs  Pack 1: retained the brand name font and positioning of descriptor; Pack 2: with brand name in standard font in prominent position and with descriptor; Pack 3: with smaller brand name in standard font and no. of cigarettes in larger font; Pack 4: pack 3 with a large graphic health warning (covering 80% of the front of the pack). All conditions displayed same graphic health warning visible on the top (packs 1-3 on 30% of the pack face, pack 4 on 80%)  Viewed 1 of 15 conditions: 5 packs (regular or 1 of 4 standardised packs) for 3 most popular Australian brands | **Appeal: Attractiveness** Positive pack ratings (‘popular brand’, ‘attractive’, ‘value for money’, ‘exclusive’, ‘brand would try/smoke’) reduced (p<0.001) as more branding elements were removed from the standardised packs.  **Appeal: Quality** Positive taste ratings (‘rich’, ‘satisfying’, ‘high quality’) were lower (p<0.001) for the standardised pack compared with the original branded pack; differences in ratings increased as more branding elements were removed from the standardised packs.  **Appeal: Smoker Identity** Positive attributes associated with each pack (‘*trendy’, ‘young’, ‘masculine’, ‘sociable’, ‘confident’*) reduced significantly (p<0.001) as more branding elements were removed from the packs. Respondents who saw the standardised pack rated smokers of that pack more *‘lower class’* (p<0.01) than did those who saw original branded pack.  **Appeal: Sub-Groups** Regardless of type of pack shown, established (regular) smokers had more favourable perceptions of all the packs (p<0.05) compared with ‘non susceptible’ non-smokers, ‘susceptible’ non-smokers and experimenting smokers. Established smokers gave more positive pack ratings (‘popular brand’, ‘attractive’, ‘value for money’, ‘exclusive’, ‘brand would try/smoke’) than any type of non-smoker for the standardised pack (*d* = 1.26, 95%CI = 0.75, 1.76).  **Health Warnings** Participants were asked to recall the health warning after viewing one pack. Comparing only standardised packs 3 and 4 (identical packs but with either 30% or 80% warnings) no effect on health warning recall was found. Overall, 58% correctly recalled the pictorial health warning and this did not vary by pack condition (p>0.10).  **Harm and Strength** Standardised brown packs perceived as less ‘lighter tasting’ (incorporating two factors: ‘be like a light cigarette’ and ‘be low in tar and nicotine’) than original branded packs. Interaction effect indicated that ratings of ‘light taste’ differed significantly (p<0.01) across pack conditions for one brand only (Longbeach). Longbeach’s standardised pack 3 and standardised pack 1 were rated as less ‘‘light tasting’’ as compared with the original branded Longbeach pack.  **Harm and Strength: Sub-Groups** No significant relationship between pack condition (standardised or original branded) with smoking status. |
| **Hammond 2009**  **Rel:** Medium  **Qual:** Medium | **Location:** UK  **Sample size:** 516 (a),  806 (b)  **Age:** mean 38.5 years (a), mean 14.6 years (b)  **Gender:** mixed  **Smoking status:** smokers only (a), mixed (b)  **Sample type:** convenience sample | To examine the effect of brand descriptors and imagery on the perceived attractiveness, taste and harm of standardised and branded cigarette packs by adults (a) and youths (b)  Survey (with experimental within-subjects design) (a), (b)  Online | Standardised packs with standardised white or brown background. Name of each brand including descriptors in Arial 14 point font.  Standardised and branded packs displayed same pictorial health warning covering 30% of pack’s lower front face.  Participants viewed pairs of leading UK cigarette brands packs on screen. Only the 4 comparisons with standardised packs are considered here: a branded pack (2 brands) vs. a white standardised pack; a branded pack (2 brands) vs. a brown standardised pack; a white standardised pack with ‘Smooth’ descriptor vs. a white standardised pack; and a brown standardised pack with ‘Gold descriptor vs. a brown standardised pack. For each brand pair, the pack positions were randomised. | **Appeal: Attractiveness** Standardised packs (both white and brown) consistently rated less attractive (p<0.001) than branded packs, by both adults and youths. Standardised packs (both white and brown) consistently rated by youths as the one they were less likely to use if they tried smoking (p<0.001) compared with branded packs.  **Appeal: Quality** Standardised brown packs rated less smooth in taste (p<0.001) than branded packs across all brands and by both adults and youths; differences in perceived taste found less often for standardised white packs compared with branded packs.  **Harm and Strength** Respondents were asked which brand had the most tar, which they would buy if they were trying to reduce the risks to their health, which would make it easier to quit smoking (adult smokers only). Response options were either pack or ‘neither/no difference’. The latter 6 out of 14 paired comparisons included standardised packs (differences significant at p<0.001 unless specified otherwise):  (a) Branded vs. white standardised pack (brand 1). Standardised rated as significantly lower tar, lower health risk, easier to quit (adults); lower tar and lower health risk (p=0.005) (youths).  Branded vs. white standardised pack (brand 2). Standardised rated as significantly lower tar, lower health risk, easier to quit (adults); lower tar (p=0.05) and no difference for health risk (youths).  (b) Branded vs. brown standardised pack (brand 1). No differences on all 3 measures (adults); no difference tar level or health risk (youths).  Branded vs. brown standardised pack (brand 2). Standardised rated as significantly higher tar (p=0.02), greater health risk (p=0.005); no difference in ease of quitting (adults). Standardised rated greater health risk (p=0.001) and no difference in tar level (youths).  (c) White standardised pack vs. same with ‘Smooth’ descriptor. ‘Smooth’ standardised pack rated as significantly lower tar, lower health risk and easier to quit (adults); lower tar and lower health risk (youths).  (d) Brown standardised pack vs. same with ‘Gold’ descriptor. ‘Gold’ standardised pack rated as significantly lower tar and lower health risk and easier to quit (adults); lower tar and lower health risk (youths). |
| **Hammond 2013^§^**  **Rel:** High  **Qual:** Medium | **Location:** UK  **Sample size:** 947  **Age:** 16-19 years  **Gender:** females  **Smoking status:** mixed  **Sample type:** convenience sample | To examine the effect of standardised cigarette packaging of female-oriented brands on female youths’ perceived appeal, taste, tar yield, health risks and smoker traits  Survey (with experimental between-subjects design)  Online | Standardised packs (condition 3) were of a mid-brown background colour, generic black font (brand name and number of cigarettes, no descriptors) and the same shape/size of container as the original.  10 packs, viewed one at a time, in 4 experimental conditions (only the comparisons with standardised packs are considered here): (1) female-oriented packs; (2) female-oriented packs with brand imagery, including colours and graphics, but with descriptors (e.g. “slims”) removed; (3) female-oriented packs without brand imagery and descriptors (standardised packs); (4) popular UK brands of ‘regular’ or non-female-oriented packs. All packs displayed same text health warning on the pack’s lower front face. | **Appeal: Attractiveness** Standardised packs rated less appealing (p<0.001) than branded packs targeted at women. Participants offered standardised packs in hypothetical selection task less likely to accept than participants offered branded packs (*χ*^2^ = 5.0, p=0.026).  **Appeal: Quality** Standardised packs rated lower in taste than branded packs targeted at women. Linear regression: branded packs rated higher in taste than standardised packs (p<0.001) and packs targeted at men (p<0.001); standardised packs rated lower in taste than packs targeted at men (p=0.027) and packs with no descriptors (p=0.013).  **Appeal: Smoker Identity** Examined smoker trait associations (‘*female’, ‘slim’, ‘glamorous’, ‘cool’, ‘popular’, ‘attractive’* and ‘*sophisticated’*) with different packs. Linear regression: branded packs given higher (p<0.001) trait scores than standardised packs.  **Appeal: Sub-Groups** Young women smokers (β=0.09, p=0.007) rated all packs, both standardised and branded, as more appealing than did non-smokers; difference not observed specifically for standardised packs. Young women who expressed greater weight concerns (β=0.11, p=0.001) more likely than women with lesser weight concerns to rate packs (both standardised and branded) as appealing in an experimental cross-sectional survey. Non-white respondents more likely than white respondents to rate packs (both standardised and branded) as appealing (β=0.11, p=0.001).  **Harm and Strength** Branded packs more likely to be rated lower tar (p=0.013) and lower health risk (p=0.004) than standardised packs.  **Harm and Strength: Sub-Groups** Smokers more likely than non-smokers to rate packs, both branded and standardised, as lower health risk (β=0.08, p<0.027) and lower tar (β=0.13, p=0.001). Non-white respondents more likely than white respondents to rate packs, both branded and standardised, lower health risk (β=0.16, p<0.001) and lower tar (β=0.13, p=0.001). |
| **Hammond 2011**  **Rel:** Medium  **Qual:** Medium | **Location:** USA  **Sample size:** 826  **Age:** 18-19 years  **Gender:** females  **Smoking status:** mixed  **Sample type:** convenience sample | To examine the effect of standardised cigarette packaging of female-oriented brands on female youths’ perceived appeal, taste, tar yield, health risks and smoker traits  Survey (with experimental between-subjects design)  Online | Standardised packs (condition 3) were of a mid-brown background colour, generic black font (brand name and number of cigarettes, no descriptors) and the same shape/size of container as the original.  8 packs, viewed one at a time, in 4 experimental conditions (only the comparisons with standardised packs are considered here): (1) branded female-oriented packs (6 USA and 2 UK popular cigarette brands); (2) branded female-oriented packs with brand imagery, including colours and graphics, but with descriptors (e.g. “slims”) removed; (3) female-oriented packs without brand imagery and descriptors (standardised packs); (4) branded non-female-oriented packs (8 USA popular cigarette brands). None of the packs displayed health warnings. | **Appeal: Attractiveness** Standardised packs rated less appealing (p<0.05) than branded packs. Linear regression: branded packs rated more appealing than standardised packs (p<0.001) and packs targeted at men (p<0.001). Standardised packs rated less appealing than packs with no descriptors (p<0.001) and packs targeted at men (p<0.001). Significantly fewer standardised packs than branded packs were selected by respondents at the end of study when given the option of taking a pack as a gift.  **Appeal: Quality** Standardised packs rated lower on taste (p<0.05) than branded packs (6 of 8 brand comparisons). Linear regression: branded packs rated better on taste than packs with no descriptors (p=0.004) and standardised packs (p<0.001); standardised packs rated lower on taste than packs targeted at men (p<0.001) and packs with no descriptors (p<0.001).  **Appeal: Smoker Identity** Examined strength of ‘smoker trait’ associations (‘*female’, ‘slim’, ‘glamorous’, ‘cool’, ‘popular’, ‘attractive’* and ‘*sophisticated’)* with different cigarette packs. Linear regression: branded packs higher (p<0.001) trait scores than standardised packs.  **Appeal: Sub-Groups** No significant differences by smoking status, age, income, education, ethnicity, or weight concerns in likelihood of selecting standardised pack at end of study. Smokers (β=0.13, p=0.001) more likely than non-smokers to endorse positive smoker traits for both branded and standardised packs. Women with greater weight concerns more likely than women with lesser weight concerns to rate packs (branded and standardised) as appealing (β=0.08, p=0.03). High-income (β=0.11, p=0.004) and high education (β=0.08, p=0.05) respondents endorsed more positive smoker traits for packs (both standardised and branded) than low income and low education respondents. Non-white respondents (β=0.10, p=0.008) more likely than white respondents to endorse positive smoker traits for both branded and standardised packs.  **Harm and Strength** Standardised packs rated lower tar (p<0.05) than branded packs (2 of 8 brand comparisons). Linear regression: branded packs more likely to be rated lower tar (p=0.004) and lower health risk (p=0.08) than standardised packs.  **Harm and Strength: Sub-Groups** Smokers more likely than non-smokers to rate branded packs lower tar (β=1.2, p<0.001) and to rate packs (both branded and standardised) lower health risk (β=0.08, p=0.05). |
| **Hoek 2009**  **Rel:** High  **Qual:** Medium | **Location:** New Zealand  **Sample size:** 245  **Age:** mean 22 years  **Gender:** mixed  **Smoking status:** mixed  **Sample type:** convenience sample | To measure perceived appeal of standardised packs, familiar and unfamiliar branded packs; and interaction of text and pictorial health warnings.  Survey (with experimental within-subjects design)  Interview method  Hypothetical scenario | Standardised pack with white background, textual health warning and pictorial health warning, brand name in standardised black font, no descriptors  Respondents asked to rate sets of 3 standardised and branded packs from ‘best’ to ‘worst’ choice in terms of attractiveness and other attributes. | **Appeal: Attractiveness** Participants 25 times more likely to give standardised pack with a pictorial health warning the ‘worst’ rating (p<0.002) than an unknown branded pack with a pictorial health warning. |
| **Hoek 2011**  **Rel:** High  **Qual:** Medium | **Location:** New Zealand  **Sample size:** 292  **Age:** mean 18-30 years  **Gender:** mixed  **Smoking status:** smokers  **Sample type:** purposive sample | To assess the effects of exposure to a standardised pack with a 75% graphic health warning measure on smokers’ perceptions of pack attractiveness.  Survey (with experimental within-subjects design)  Interviews  Hypothetical scenario | Standardised pack with a 75% graphic health warning relative to a branded pack with a 30% graphic health warning. Standardised packs varied in amount of original branding elements and information retained.  Respondents asked to rate sets of 4 standardised and branded packs from ‘best’ to ‘worst’ choice in terms of attractiveness and other attributes. | **Appeal: Attractiveness** As pack branding elements were reduced, choice of these packs as ‘best’ declined and those chosen as ‘worst’ increased. Least branded packs significantly (p=0.000) less likely to be chosen than fully branded options. |
| **Munafò 2011**  **Rel:** High  **Qual:** Medium | **Location:** UK  **Sample size:** 43  **Age:** 21-28 years  **Gender:** mixed  **Smoking status:** mixed  **Sample type:** convenience sample | To assess the impact of standardised packaging on visual attention towards health warning information on cigarette packs  Survey (with experimental between-subjects design)  Observation to track eye movements (saccades)  Measured saccades towards images of branded and standardised packs presented on a computer screen. | White with brand name in black standardised font and combination of ten pictorial warnings used on pack front  Ten different randomly selected UK pictorial health warnings were paired with each of 10 branded and 10 standardised packs, to create a total of 200 images. Participants saw 20 images randomly selected from this set, using 10 different health warnings, each presented once on a branded pack and once on a standardised pack. | **Health Warnings** Standardised packs, in comparison to branded packs, lead to more saccades (eye movements) towards the warnings among non-smokers (p=0.001) and weekly smokers (p=0.001). This effect was not observed for daily smokers (p=0.35).  **Health Warnings: Sub-Groups** as above. |
| **Swanson 1997**  PhD thesis  **Rel:** High  **Qual:** Medium | **Location:** Australia  **Sample size:** 301  **Age:** 14-17 years  **Gender:** mixed  **Smoking status:** smokers  **Sample type:** convenience sample | To measure the effect of current and standardised packaging on ratings of smoker identity by young smokers  Survey (with experimental between-subjects design)  Interviews  Hypothetical scenario | Standardised packs with white background, with health warning and descriptors, brand name in standardised black lettering  Respondents were shown a series of packs, each accompanied by two images of people (one consistent with the brand, such as an outdoor man for the brand Marlboro, one inconsistent with the brand, such as a young woman for a brand typically smoked by older people), and asked if the pack was *‘right or wrong’* for the person. | **Appeal: Smoker Identity** When standardised packs were shown instead of branded packs, respondents’ positive associations of brands with the ‘*right*’ sort of person for the brand weakened, as did negative associations with the ‘*wrong*’ sort of person. Differences significant for 4 of 6 comparisons (p<0.01 – p<0.001). |
| **Thrasher 2011**  **Rel:** Medium  **Qual:** Medium | **Location:** USA  **Sample size:** 402  **Age:** mean 38 years  **Gender:** mixed  **Smoking status:** smokers  **Sample type:** convenience sample | To estimate differences in demand associated with different health warning label formats and standardised packaging with smokers  Survey (with experimental between-subjects design)  Hypothetical scenario: individuals asked to state the amount of money they would ‘bid’ for different types of cigarette pack | Standardised beige pack with graphic and text warnings of mouth cancer, covering 50% of the lower half of the front, back and one side of the package. All colour and symbolic brand elements removed.  4 types of pack for 3 brands: fully branded with 50% text warning on one side; branded with 50% text warning on front, side and back; branded with 50% picture and text warning; and standardised with 50% picture and text warning.  Respondents asked to bid 3 times for selected pack, the final bid was binding. | **Appeal: Attractiveness** Mean average of bids decreased across conditions (Branded pack, minimal text warning 1: US$3.52; Branded pack, larger text warning 2: US$3.43; Branded pack, picture and larger text warning 3: US$3.11; Standardised pack, picture and larger text warning 4: US$2.93). Demand was significantly lower for both packs with prominent pictorial warnings (3 and 4), with the lowest demand associated with the standardised pack. Results from both bivariate (p<0.01) and multivariate models (p<0.01) indicate statistically significant lower bids for standardised, unbranded pack with pictorial warning compared with branded pack with pictorial warning. |
| **Wakefield 2008**  **Rel:** High  **Qual:** Medium | **Location:** Australia  **Sample size:** 813  **Age:** 18-30+ years  **Gender:** mixed  **Smoking status:** smokers  **Sample type:** national non-representative sample | To examine the effect of progressively more ‘standardised’ packaging on perceptions of pack appeal, brand imagery characteristics and inferred smoking experience  Survey (with experimental between-subjects design)  Online | Three standardised brown cardboard packs becoming progressively standardised: 1) maintains branded font and positioning of brand/descriptor; 2) with brand name in standard font in prominent position on the pack with descriptor information in standard font at bottom; and 3) with brand name in smaller standard font positioned at bottom with number of cigarettes in larger font in prominent position on the pack. All displayed graphic health warnings on upper 30% of pack face.  Respondents randomly shown one pack image from a possible 12 packs (3x4 design: 3 brands, 4 different types of packaging – original brand plus three standardised packs). | **Appeal: Attractiveness** Logistic regression: standardised pack which preserved brand name font, placement and brand variants rated as less attractive (p<0.01) than original branded pack; difference increased (p<0.001) as progressively more brand elements were removed.  **Appeal: Quality** Bivariate logistic regression analysis: as packs became progressively standardised, they were rated as less rich in tobacco flavour (p<0.05), and the standardised pack with the least branding elements also rated as less satisfying (p<0.05) and less likely to be of the highest quality tobacco (p<0.05).  **Appeal: Smoker Identity** Smokers of standardised packs which preserved brand name, font, placement and brand variants perceived as less *sociable/outgoing* (p<0.01 – p<0.001) and *trendy/stylish* (p<0.10 – p<0.001) than smokers of original branded packs; negative perceptions increased as progressively more brand elements were removed, with smokers of the standardised pack with the least branding elements also rated less *mature* (p<0.05) and less *masculine* (p<0.01).  **Harm and Strength** Fewer smokers who viewed standardised packs 2 and 3 rated them low in tar and nicotine (p<0.05) compared with those who viewed original branded pack. |
| **White 2011**  MSc thesis  **Rel:** High  **Qual:** Medium | **Location:** Brazil  **Sample size:** 640  **Age:** 16-26 years  **Gender:** female  **Smoking status:** mixed  **Sample type:** national representative sample | To examine the effect of standardised cigarette packaging and brand descriptors on female youths’ perceived appeal, taste, tar yield, health risks and smoker traits  Survey (with experimental between-subjects design)  Online | Standardised packs (conditions 2 and 3) of mid-brown background colour, brand name in generic black font, and the same shape/size of container as the original: one standardised pack with descriptors, one standardised pack without descriptors.  (a) “Individual pack ratings” 10 packs, one at a time, in 3 experimental conditions: (1) branded packs (leading Brazilian and international cigarette brands); (2) standardised pack with descriptors; (3) standardised pack without descriptors. None of the packs displayed health warnings.  (b) “Direct pack comparison” 10 packs in comparison pairs (2 packs – a “lighter” and “regular” brand – from each of 5 brand families), in 2 experimental conditions: as (1) and (2) above. | **(a) Appeal: Attractiveness** Packs rated less appealing as more elements of branding, including descriptors, removed. Compared with branded packs, standardised packs rated as less appealing (p<0.01) and less desirable (p<0.01) to be seen smoking with. In hypothetical pack selection task, 75% chose branded packs vs. 25% a standardised pack.  **Appeal: Quality** Standardised packs rated poorer tasting (p<0.001) and less smooth (p<0.05) than branded packs; difference in rating increased as descriptors were removed from the standardised packs.  **Appeal: Smoker Identity** Smokers image ratings (*female/male, stylish/not stylish, popular/not popular, sophisticated/not sophisticated,* and *slim/overweight*) significantly lower for standardised than branded packs for 3 traits, ‘*female’* (p<0.05), *‘stylish’* (p<0.05) and *‘sophisticated’* (p<0.05). Linear regression: standardised packs (p=0.001) and standardised packs with no descriptors (p<0.001) less likely to be rated ‘*female’* than branded counterparts. Standardised packs with no descriptors less likely to be rated ‘*female’* than other standardised packs (p<0.001).  **Appeal: Sub-Groups** No significant differences in appeal ratings by smoking status, age, education or ethnicity.  **(b) Harm and Strength** Branded packs rated as less harmful than standardised packs in individual ratings. In paired comparisons, regardless of condition (standardised or branded), ‘lighter’ pack (as designated by the researchers according to descriptor information) consistently rated less harmful and easier to quit than ‘regular’ pack. Standardised packaging did not impact on proportion of participants who perceived ‘no difference’ between pack pairs in terms of health risk or ease of quitting.  **(a) Harm and Strength: Sub-Groups** Smokers rated all packs, both standardised and branded, less harmful (p=0.019) than did non-smokers. Older participants less likely than younger (p=0.007) to perceive differences in harmfulness between packs, regardless of whether branded or standardised. Non-white respondents more likely than white respondents to perceive some packs, regardless of whether branded or standardised, as less harmful (p=0.008). |
| ***Cross-sectional surveys without an experimental design (*n *= 3)*** | | | | |
| **Beede 1990**  **Rel:** High  **Qual:** Medium | **Location:** New Zealand  **Sample size:** 567  **Age:** 12-14 years  **Gender:** mixed  **Smoking status:** mixed  **Sample type:** convenience sample | To investigate adolescents’ attitudes towards standardised packaging and its potential impact on smoking behaviour, and to measure the impact of packaging design on health warning recall.  Survey  Interview method  Focus groups | Standardised packs with white background and black printing – individual brand names retained. Size, type face, health warnings, pack size and tar and nicotine contents consistent across all standardised packs.  Recall of pack elements on both standardised and branded packaging. | **Health Warnings** Higher recall of health warnings, overall, on standardised packs (74.0%) than branded packs (63.8%). Recall of warnings on standardised packs, compared to branded packs, was always higher, although only significantly so for the second (p=0.03) and third (p=0.001) packs recalled. Recall for both branded and standardised packs was higher for familiar packs from New Zealand (79.4% branded; 82.3% standardised) than for unfamiliar packs from the USA (45.2% branded; 65.0% standardised). |
| **Bondy 1996**  **Rel:** Low  **Qual:** High | **Location:** New Zealand  **Sample size:** 14,270  **Age:** 10-19 years  **Gender:** mixed  **Smoking status:** unclear  **Sample type:** national representative sample | To investigate access to tobacco and awareness of tobacco marketing and branding, including brand recognition and attractiveness, among youth.  Survey  Questionnaires | ‘Generically packaged cigarettes’ in uniform colour (no further details)  Respondents provided a hand-out with drawings of 1 generic [standardised] and 9 branded cigarette packages with the brand names masked and asked to pick the “most attractive” and “least attractive” packages. | **Appeal: Attractiveness** 71% of participants ranked single standardised pack presented (amongst 9 branded packs with their brand names masked) as least attractive (significance value not given). The study focused on a wide range of issues related to access to tobacco, knowledge of brands and awareness of tobacco advertising and sponsorship. (Packaging was just one element and thus the study is rated as low relevance.) |
| **Moodie 2012**  **Rel:** High  **Qual:** Medium | **Location:** UK  **Sample size:** 658  **Age:** 10-17 years  **Gender:** mixed  **Smoking status:** mixed  **Sample type:** convenience sample | To examine the role of packaging in choice of cigarettes, perceptions of pack colours and strength and harm of different standardised packs  Survey  Online | Standardised (flip-top) packs with 4 different background colours – green, red, light blue and white – and same text health warning  Plus ‘dark brown’ standardised packs with same text health warning in 3 different shapes/opening styles (flip-top (regular shape), slide and superslims).  No comparison with branded packs | **Appeal: Attractiveness** Standardised brown flip-top pack rated ‘unattractive’ (91%), ‘uncool’ (87%) and ‘a pack you would not like to be seen with’ (88%).  **Appeal: Quality** Lighter coloured flip-top standardised packs (white and light blue) associated with weaker taste.  **Appeal: Smoker Identity** Standardised brown flip-top pack associated with an *‘unpopular’* (59%), ‘*unfashionable’* (67%), ‘*boring’* (63%) and ‘*old’* (69%) person.  **Appeal: Sub-Groups** Smokers less negative than never-smokers on 6 of 8 appeal items (p<0.01 – p<0.001). Ever-smokers significantly less likely than never-smokers to rate standardised brown flip-top packs ‘unattractive’ (*OR* = 0.36, 95%CI = 0.21, 0.63)*. Non-susceptible never-smokers more likely than susceptible never-smokers to report that they would not like to be seen with the standardised brown flip-top pack (p<0.01). Non-susceptible never-smokers more likely to perceive a person smoking from a standardised brown flip-top pack more negatively than susceptible never-smokers on all 4 measures used (p<0.001).  **Harm and Strength** Just under half made an association between harm and colour (44% made associations between pack colour and most harmful cigarettes, 46% between pack colour and least harmful cigarettes, with 56% and 54% respectively making no association between harm and colour). Red pack rated most harmful by 22%. Green pack rated most harmful by 12% and least harmful by 11%. Light blue pack rated least harmful by 15% and white pack least harmful by 18%.  **Harm and Strength: Sub-Groups** Ever-smokers more likely to associate colour with harm than never-smokers (p<0.01). Susceptible never-smokers more likely to make associations between colour and harm compared with non-susceptible never-smokers (p<0.01). Where an association with colour was made, pattern was similar for never- and ever-smokers and for susceptible and non-susceptible never-smokers: red greater harm; light blue and white, less harm. |
| **Mixed method studies† (*n* = 3)** | | | | |
| **Centre for Health Promotion 1993**  **Survey**  **Rel:** Medium  **Qual:** Medium | **Location:** Canada  **Sample size:** 129  **Age:** 12-17 years  **Gender:** mixed  **Smoking status:** mixed  **Sample type:** regionally representative sample | To assess the impact of standardised packaging on the imagery projected by the cigarette package among adolescents  Survey  Questionnaires | Buff colour standardised packages had black printing in standard font. Only the brand names, health warning label appeared on these packages. All packages carried the warning C*igarettes Cause Fatal Lung Disease*, covering the top 25% of the package front and back, with a black border around it, and black lettering on a white background. Included the plastic wrap and “Canada duty paid” band.  Respondents asked to rate brand and standardised packages for two cigarette brands (four rotation orders used). | **Appeal: Attractiveness** Standardised packs rated more negatively (p<0.001) than brand equivalent packs by both smokers and non-smokers on all attractiveness ratings (ugly/attractive, boring/exciting, old-fashioned/modern, awful/nice, dull/colourful, nerdy/cool).  **Appeal: Quality** Standardised packs rated as more ‘cheap-looking’ (p<0.001) than brand equivalent packs by both smokers and non-smokers.  **Appeal: Smoker Identity** Standardised packs rated more appropriate (p<0.001) to *‘old people’* than *‘young people’* by both smokers and non-smokers, even where the standardised pack brand was one favoured by young people.  **Appeal: Sub-Groups** Findings reported separately for smokers versus non-smokers on attractiveness of standardised packs, but insufficient data reported to calculate significance or effect sizes. |
| **Goldberg 1995**  **Survey** (a)  **Rel:** High  **Qual:** Medium  **Survey** (b)  **Rel:** Medium  **Qual:** Low | **Location:** Canada  **Sample size:** 1200 (a), 400 (b), 100 (c)  **Age:** 14-17 years (a),  12-14 years (b)  **Gender:** mixed **Smoking status:** mixed  **Sample type:** regionally representative sample (a), convenience sample (b) | To assess the impact of standardised and standardised packaging of cigarettes on recall of health warning messages and on ratings of smoker identity  Survey (with experimental between-subjects design) (a, b)  Survey administered in focus groups (b) | Two standardised packs in standardised white box with all brand markings removed except for the name. Both with text health warning, one with and one without pictorial “lungs” health warning.  (a) Smoker Identity (“Visual Image Experiment”): 6 pack images for one of three brands; each brand had 2 person/image types, one consistent and one inconsistent with the brand; each brand had 3 pack types (branded, standardised or standardised with lung image).  (b) Health Warnings (“Recall & Recognition Experiment”): Participants shown images of 4 items including a cigarette pack, and asked to recall all the information they could, both unaided and prompted. Half saw 3 cigarette brands in their branded packs, and half saw the 3 brands in standardised packs. All packs had only text health warnings and each brand had a different warning. | **(a) Appeal: Smoker Identity** For all three Canadian brands, removal of brand markings led respondents to view pack as less appropriate for the person image which was normally associated with the brand. (Overall, the standardised pack scored a significantly lower mean appropriateness score out of 5 (mean=2.73) than the branded pack (mean=3.87; F=638.59, p≤0.0001).)  **(b) Health Warnings** Analyses of the results are reported in two papers, the 1995 Health Canada research report and the 1999 article in the *American Journal of Public Health*, with differences in the direction of effect found. As reported in the 1995 report, in the unaided recall exercise, recall for the ‘Smoking can kill you’ warning was 38% for the branded pack and 50% for the standardised pack (p<0.05). However, there were very low levels of unaided recall for ‘Cigarettes are addictive’ and ‘Tobacco smoke causes fatal lung disease in non-smokers’ (4% and 3% respectively, compared to the 44% unaided recall rate for the ‘Smoking can kill you’ warning), which prohibited meaningful analysis. A contrary result was that aided recall of health warning message was higher when displayed on branded packs than on standardised packs. Participants were more likely to match the correct warning message to the branded pack than to the standardised pack (p<0.0001), for two of the three warnings: ‘Smoking can kill you’ (55% branded versus 42% standardised) and ‘Tobacco smoke causes fatal lung disease for non-smokers’ (58% branded versus 46% standardised). Aided recall of ‘Cigarettes are addictive’ was “close to 50%” for both standardised and branded cigarettes.  The analysis of the data published in 1999 reported that recall for two warnings favoured the standardised pack: the ‘Smoking can kill you’ warning was 22% for the branded pack and 56% for the standardised pack (p<0.001) and recall for the ‘Cigarettes are addictive’ warning was 13% for the branded pack and 27% for the standardised pack (p=0.06). Whereas recall of the ‘Tobacco smoke causes fatal lung disease in non-smokers’ warning, favoured the branded pack, at 1% for the standardised pack and 15% for the branded (p<0.05). |
| **Rootman 1995**  **Survey**  **Rel:** High  **Qual:** Medium | **Location:** USA and Canada  **Sample size:** 2132  **Age:** 12-14 years  **Gender:** mixed  **Smoking status:** mixed  **Sample type:** regionally representative sample | To examine the impact of standardised packaging on recall of health warning information, and the impact of changes in price on youth smoking  Survey | Two standardised packs, white and off-white (no further details).  Perceptions study: viewed posters of one standardised and one branded pack in classroom survey from 6 variations: 2 brands x 3 pack types (regular, white and off-white).  Warnings recall study: viewed one poster of a pack for 1 minute in classroom survey from 6 variations: 2 brands x 3 pack types (regular, white and off-white).  Both studies collapsed the 6 variations into 2 categories for analysis, regular and standardised packs. | **Appeal: Attractiveness** Majority stated that they preferred and would rather take home a branded pack than a standardised pack. Canadian respondents described standardised packs as ‘more boring’ than regular packs (86% vs. 5%) and ‘uglier’ (78% vs. 8%) (significance values not given).  **Appeal: Smoker Identity** Branded pack associated with ‘*cool kids*’ by 64% of Canadian respondents, compared with only 5% for standardised pack (significance values not given).  **Health Warnings** Canadian respondents almost twice as likely to report that it was easier to see health warnings on standardised (51%) than branded pack (29%). Recall of warning was improved by standardised packaging, but only for regular smokers; 82% of daily smokers remembered warning on standardised pack, 62% for branded pack (p<0.05). No differences in warning recall on standardised or branded packaging among USA sample. 53% of participants in Canada said that the standardised pack made the health warning look more serious, compared to 19% who said that the branded pack made it look more serious. |
| **Intervention studies‡ (*n* = 1)** | | | | |
| **Moodie 2011**  **Intervention**  **Rel:** High  **Qual:** Medium | **Location:** Scotland  **Sample size:** 48  **Age:** 18-35 years  **Gender:** mixed **Smoking status:** smokers  **Sample type:** random quota sample | To explore the impact of standardised cigarette packs in real-life settings among young adults  Intervention: randomised study | Dark brown standardised pack with fictitious brand name in black standardised font and text warning *Smoking Kills* on pack front and pictorial warning (diseased and healthy lungs) on reverse panel.  Smokers asked to transfer their cigarettes from their own packs to standardised packs provided and use these standardised packs for two weeks, either at the beginning or end of the four week experimental period, and use their own packs for the other two weeks of the study. | **Appeal: Attractiveness** Standardised brown pack rated more negatively than usual branded pack in terms of stylishness (p<0.001), fashionability (p<0.01), coolness (p<0.001), attractiveness (p<0.001) and appeal (p<0.001).  **Appeal: Quality** Standardised brown packs rated less expensive (p<0.001) and poorer quality (p<0.001) than usual branded pack.  **Health Warnings** Warnings more noticeable on standardised packs (p<0.05) (1^st^ and 2^nd^ measure). Warnings rated more serious on standardised packs (p<0.001) (4^th^ measure). No differences in believability of warnings between branded and standardised packs. Overall warnings ratings did not differ between branded and standardised packs and did not vary across time.  **Harm and Strength** No differences between standardised and branded packs in ratings of pack making you aware of health risks. |

**Notes to table:** *Odds ratio and 95% confidence interval calculated by the review team and not printed in the study’s paper. †Only the quantitative elements and not the qualitative elements of the mixed methods studies are reported in this paper. ‡Only the quantitative elements and not the qualitative elements of the intervention study are reported in this paper. **^§^**The study’s publication date has been revised since the original source review [19] was published.
